# Salt and Nutritional Content of Foods Advertised During Televised Professional Football Games

**DOI:** 10.1001/jamanetworkopen.2024.57307

**Published:** 2025-01-30

**Authors:** Lara Al-Zoubaidi, Nadya Vinsdata, R. Eric Heidel, Paul J. Hauptman

**Affiliations:** 1 Department of Nutrition, Saint Louis University, St Louis, Missouri; 2 University of Nevada, Reno School of Medicine, Reno; 3 Department of Surgery, University of Tennessee Graduate School of Medicine, Knoxville

## Abstract

This cross-sectional study assesses the nutritional content of foods advertised during televised professional football games.

## Introduction

Modest restriction of dietary sodium intake for individuals with heart failure (HF), including those with concomitant kidney failure and advanced disease, may improve symptoms and quality of life.^1^ A registered dietitian- or nurse-coached intervention with 2 to 3 g/d sodium restriction improved New York Heart Association functional class and leg edema in individuals with HF with reduced ejection fraction.^2^ However, excess dietary intake is frequent with ingestion of processed and prepared foods, including those from high-volume restaurants or other food establishments. Additionally, excess fat and caloric intake may influence the natural history of coronary artery disease, diabetes, and other conditions. Since advertising by the food and restaurant industry influences consumer behavior and meal option selections,^3^ we analyzed advertisements transmitted during televised professional football games in the US to assess the nutritional content by serving.

## Methods

This cross-sectional study did not meet the definition of human participants research and therefore institutional review board review was not required. This study is reported following the Strengthening the Reporting of Observational Studies in Epidemiology (STROBE) reporting guideline.

Using a cross-sectional design, we recorded a convenience sample of 10 televised National Football League (NFL) games from September to November 2023 and analyzed commercials involving food (store-bought foods and quick-service restaurants), including the duration and frequency of the advertisement. Individual serving size was determined by the presentation in each commercial (eg, a medium-sized pizza or fried chicken sandwich). The nutritional content (fat, cholesterol, sodium, sugar, protein, and carbohydrates) of each food item was determined from each company’s website. We used Dietary Reference Intakes, the Dietary Guidelines for Americans 2020-2025, and the American Heart Association to compare with recommended daily intakes (eMethods in Supplement 1). Medians and IQRs were reported, since all continuous variables did not meet normality. Data were analyzed using SPSS software version 29 (IBM) on August 4 and 5, 2024.

## Results

A total of 102 food-related commercials (median [IQR] duration, 15 [15-20] seconds) from 18 vendors were assessed, representing 10.8% of all consumer-facing advertiser content. The median (IQR) sodium content was 910 (220-1872) mg; other nutritional content is shown in Table 1 and Table 2. Meals at quick-service restaurants were highest in sodium, carbohydrates, and calories compared with smaller store-bought food options.

**Table 1.** Nutritional Content of Advertised Foods, by Percentile

Variable	Percentile			Recommended dietary intakes^a^
	25th	Median (50th)	75th	
Calories	160	490	870	2000 kcal (females) and 2600 kcal (males)
Fat, g	8	14	50	Saturated fat: <10% of kcal/d
Cholesterol, mg	0	35	85	None
Sodium, mg	220	910	1872	2300 mg
Sugar, g	5	11	15	<10% total kcal/d or 24 g added sugar (female) and 36 g added sugar (male)
Protein, g	2	14	44	46 g (females) and 56 g (males)
Carbohydrates, g	19	56	80	130 g

^a^ Sources of recommended dietary intakes are listed in the eMethods in Supplement 1.

**Table 2.** Nutritional Content and Frequency of Advertising by Vendor

Vendor	Ads, No.^a^	No. of different servings	Duration, s	Kilocalories	Fat, g	Cholesterol, mg	Sodium, mg	Sugar, g	Protein, g	Carbohydrates, g
Store bought foods										
Hershey’s	7	1	20	160	9	5	25	18	2	19
Honey Bunches of Oats	3	2	20	160	2	0	190	9	3	34
Old El Paso enchilada	7	1	25	107	3	0	567	1	2	18
Pillsbury	8	1	15	90	3	0	220	15	2	13
Reese’s	3	1	15	87	5	1	71	8	2	9
Snickers	5	1	15	250	12	5	125	28	4	32
Tostitos	3	2	15	140	7	0	120	0	2	19
Tostitos, Lays, Doritos	4	3	30	490	28	0	540	2	5	56
Quick-service restaurant or other food establishment										
Burger King	6	4	17.5	1284	55	110	1518	59	44	154
Domino’s	9	5	15	220	11	18	220	1	5	27
Jersey Mike’s Subs	8	1	15	770	41	73.5	1872	6.4	43.6	58
Little Caesars	11	2	15	1370	76	90	2820	12	60	163
Panera Bread	6	1	15	1410	76	225	2560	12	81	107
Papa John’s	7	1	15	350	14	35	910	5	14	41
Subway	7	1	15	540	8	50	1640	12	42	80
Taco Bell	4	1	30	690	37	50	1280	4	21	64
Wingstop	4	1	15	610	50	80	1720	10	32	66

^a^ Number of repeated commercials.

## Discussion

This cross-sectional study found that the foods advertised during NFL games, the most watched sporting events in the US,^4^ were frequently high in sodium, calorie, and fat content. Although the effectiveness of sports advertising and paid sponsorships on food consumption has been studied mostly among children and young adults,^3,5,6^ adults with prevalent conditions, such as HF, coronary artery disease, hypertension, diabetes and kidney failure, may also be vulnerable to deviations from suggested or prescribed dietary restrictions based on the frequency of exposure to advertising. Furthermore, television watching is a sedentary activity, and since NFL games are televised on 3 days of the week during the regular season, the combination of extended viewing times and poor dietary choices may be maladaptive for both primary and secondary prevention of multiple chronic cardiovascular and other conditions.

Our study may be limited by the fact that we determined the nutritional content of the advertised food, but the actual intake likely varies considerably by individual. Recommended dietary intakes are established for healthy individuals as opposed to those with HF or other concomitant conditions. Some of the football games were regionally broadcast; thus, we cannot ascertain whether all the food advertisements had a national reach; additionally, we did not sample across the entire season to determine whether there are differences in early vs late season advertising content. We also did not assess other sports programming and therefore cannot determine whether there are differences in food advertising content by sport. The findings of this cross-sectional study suggest that individuals with HF and other cardiac and kidney diseases who might respond to dietary interventions should be counseled by clinicians about limiting dietary intake of most foods advertised during professional football games.

## Notes

Supplement 1.
eMethods.

Supplement 2.
Data Sharing Statement
