# Supplementary material for: Salt and Nutritional Content of Foods Advertised During Televised Professional Football Games
Source: JAMA Netw Open. 2025 Jan 30;8(1):e2457307. doi: 10.1001/jamanetworkopen.2024.57307 (PMC11783189; doi:10.1001/jamanetworkopen.2024.57307)
Supplement: Supplement 1. — eMethods. [file jamanetwopen-e2457307-s001.pdf]

Supplemental Online Content

Al-Zoubaidi L, Vinsdata N, Heidel RE, Hauptman PJ. Salt and nutritional content of foods advertised during televised professional football games. *JAMA Netw Open.* 2025;8(1):e2457307. doi:10.1001/jamanetworkopen.2024.57307

eMethods.

This supplemental material has been provided by the authors to give readers additional information about their work.

© 2025 Al-Zoubaidi L et al. *JAMA Network Open.*

eMethods. Sources for Recommended Dietary Intakes
Sources: Dietary Reference Intakes (carbohydrates, protein): https://ods.od.nih.gov/HealthInformation/nutrientrecommendations.aspx#databases. American Heart Association (sugar): https://www.heart.org/en/healthy-living/healthy-eating/eat-smart/sugar/added-sugars. Dietary Guidelines for Americans 2020-2025 (calories, sodium, fat, sugar): https://www.dietaryguidelines.gov/sites/default/files/2021-03/Dietary_Guidelines_for_Americans-2020-2025.pdf. Estimated calorie needs are based on age and actively level; the value chosen is for a moderately active middle-aged male or female.

© 2025 Al-Zoubaidi L et al. *JAMA Network Open*.
