# Supplementary material for: Salt and Nutritional Content of Foods Advertised During Televised Professional Football Games
Source: JAMA Netw Open. 2025 Jan 30;8(1):e2457307. doi: 10.1001/jamanetworkopen.2024.57307 (PMC11783189; doi:10.1001/jamanetworkopen.2024.57307)
Supplement: Supplement 2. — Data Sharing Statement [file jamanetwopen-e2457307-s002.pdf]

# Data Sharing Statement

Al-Zoubaidi. Salt and Nutritional Content of Foods Advertised During Televised Professional Football Games. *JAMA Netw Open*. Published January 30, 2025.
doi:10.1001/jamanetworkopen.2024.57307

**Data**
**Data available:** Yes
**Data types:** Data (not involving human participants)
**How to access data:** Data requests should be sent to: phauptman@med.unr.edu
**When available:** With publication

**Supporting Documents**
**Document types:** Other (please specify)
**Additional Information:** Raw data
**How to access documents:** Data requests should be sent to: phauptman@med.unr.edu
**When available:** With publication

**Additional Information**
**Who can access the data:** researchers whose proposed use of the data has been approved
**Types of analyses:** Researchers whose proposed use of the data has been approved
**Mechanisms of data availability:** After approval of a proposal,
**Any additional restrictions:** None
